# Intraoperative femoral condyle fracture in primary total knee arthroplasty - a case-control study in Asian population

**DOI:** 10.1186/s43019-020-00043-6

**Published:** 2020-06-15

**Authors:** Yik-Fung Mak, Qunn-Jid Lee, Wai-Yee Esther Chang, Yiu-Chung Wong

**Affiliations:** Department of Orthopaedics and Traumatology, Yan Chai Hospital, 7-11 Yan Chai Street, Tsuen Wan, New Territories Hong Kong

**Keywords:** Complication, Fracture, Femur, Condyle, Knee, Arthroplasty

## Abstract

**Purpose:**

Intraoperative femoral condyle fracture is a significant but rarely reported complication during primary total knee arthroplasty (TKA). This is the first study to identify the incidence, risk factors, location and outcome of these fractures in an Asian population with modern posterior-stabilized (PS) TKA.

**Materials and methods:**

We reviewed 2682 consecutive primary TKAs performed between 2011 and 2017 in a single centre; 23 femoral condyle fractures were identified and analysed.

**Results:**

Fractures were managed conservatively with screw fixation or revision arthroplasty. Mean follow up was 3.0 years (range 3 months to 5.9 years). All patients achieved bone union and good functional outcome. The mean Knee Society Knee score was 89.4 (range 71–100) and the function score was 80.2 (range 60–95) at a mean of 3.0 years post-operation. Bilateral surgery was found to be a significant risk factor for femoral condyle fracture, while there was a higher trend of fracture in female patients and Stryker articular surface mounted (ASM) navigation.

**Conclusions:**

Intraoperative fracture is not uncommon with modern PS TKA. Postulated risk factors for fracture were discussed. Early identification of risk factors and a rigorous surgical technique may reduce risk of fracture. A good functional result was expected after proper treatment.

## Introduction

Intraoperative fracture is a significant but rarely reported complication during total knee arthroplasty (TKA). Studies have reported incidence ranging from 0.39% to 2.2%, mostly occurring at the femoral condyle or tibial plateau [1–3]. With the demand for TKA increasing worldwide [4, 5], rare complications are more frequently encountered in our daily clinical management. However, clinical data has so far been lacking on identifying major causative factors of the occurrence of fractures during TKA. Moreover, there have been no studies in Asian people in particular, whose smaller body build is postulated to contribute to a specific risk of femoral condylar fracture, related to excessive box cut of the femoral component in posterior-stabilized implants and in patients with weak bone [1]. This study attempts to identify the incidence, risk factors, location and outcome of these fractures in our population.

## Materials and methods

We reviewed 2682 consecutive primary TKAs performed between 2011 and 2017 in our institute. Patient information was retrieved using the Clinical Data Analysis and Reporting System (CDARS) by Hospital Authority (HA), Hong Kong. Operative details, outpatient notes and postoperative radiographical images were accessed by the HA using the Clinical Management System (CMS) and Operative Theatre Management System (OTMS). We recorded data including demographics, site of fracture, type of implant, treatment and outcome.

All TKAs were performed by a standard anterior midline incision, with the capsule entered by medial parapatellar approach. The femur was prepared first, followed by the tibia. Osteophytes at the posterior femoral condyle were routinely removed using an osteotome. The patella was selectively resurfaced according to the surgeon’s discretion. Bone cuts were assessed using trial implants prior to the final component implantation. Cementation was applied at the femoral, tibial and patellar components.

There were 231 cases (9.4%) performed as bilateral surgery in a single operative session, while the remainder were performed as unilateral surgery. There were 772 men (31.5%) and 1679 women (68.5%). Mean age was 69.3 years (range 41–90). Mean body mass index (BMI) was 27.7 (range 14.0–59.7). Posterior-stabilized implants (*n* = 2618) or medial pivot implants (*n* = 74) were used. Computer navigation assistance was used in 816 TKAs (30.4%) according to the surgeon’s decision.

Statistical models used included the chi-square (χ^2^) test, *t* test and Fisher’s exact test. A *p* value <0.05 was considered to be statistically significant.

## Results

We identified 28 intraoperative fractures, giving an incidence of 1.04%. Amongst these cases there were 23 femoral condyle fractures (17 medial condyle, 6 lateral condyle), 3 medial tibial plateau fractures, 1 tibial tuberosity fracture and 1 patella fracture. All patients were still alive with mean follow up of 3.0 years (range 3 months to 5.9 years).

**Fig. 1:**
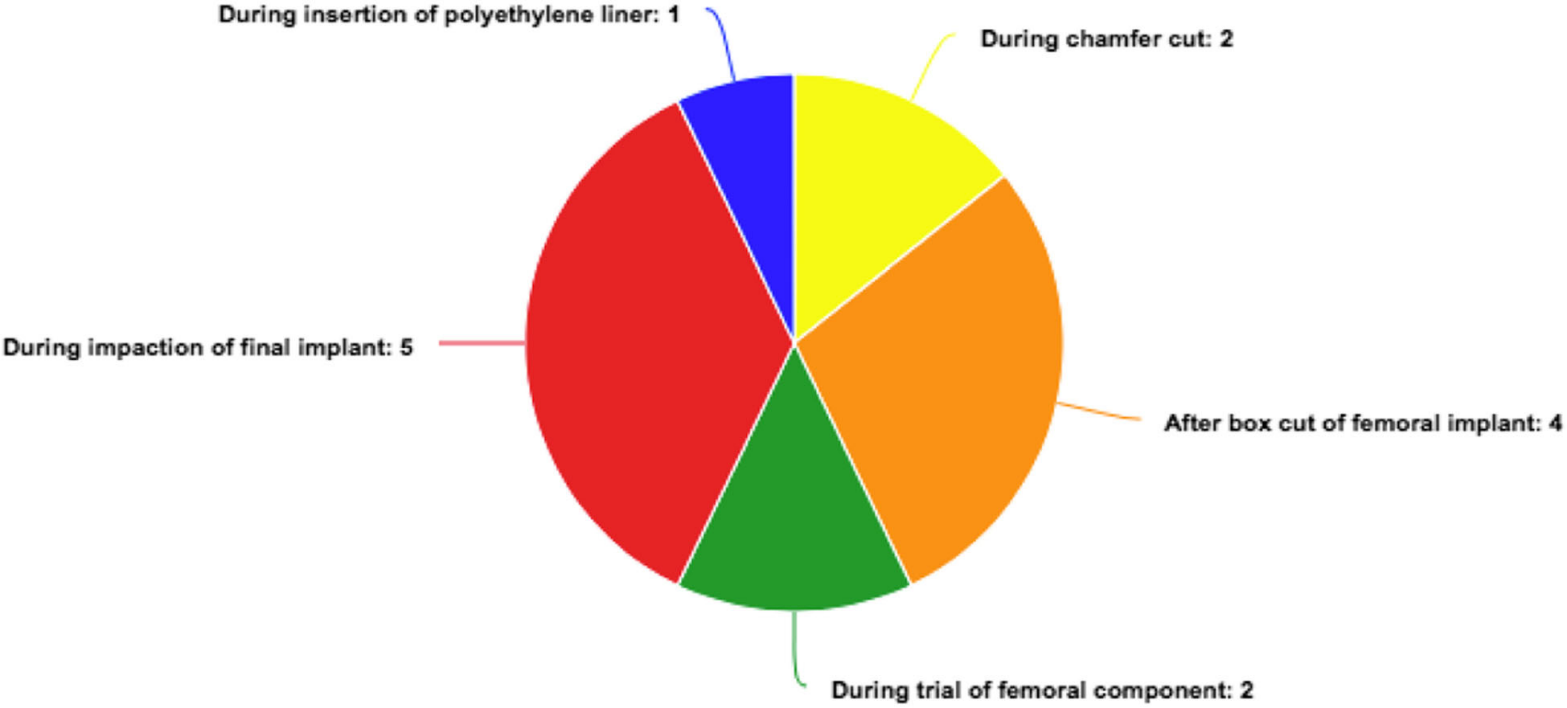
Step at which fracture occurred

The mean age of the patients with femoral condyle fractures identified (*n* = 23) (Table 1) was 69.4 years (range 56–85) at the time of surgery: there were 4 men and 18 women, and 11 left knee and 12 right knee fractures. One patient had bilateral medial condyle fractures in the same operating session. Mean BMI was 25.9 (range 20.5–34.0). There were 14 patients (63.6%) with at least one factor indicating high risk of osteoporosis [6]; 2 patients had concomitant chronic renal failure and 2 patients had history of an osteoporotic fracture. None of the patients had previously undergone knee surgery. No anterior cortical notching was noted in any patients. There were 22 fractures occurring with posterior-stabilized implants (11 with the Triathlon® Knee System by Stryker, 9 with the NexGen® Legacy® Posterior Stabilized (LPS) Flex Fixed Bearing Knee by Zimmer, 1 with the P.F.C.® Total Knee System and 1 with the ATTUNE® Knee System by DePuy Synthes) and 1 fracture with a medial pivot implant (EVOLUTION™ Medial-Pivot Knee System by MicroPort). There were 10 fractures (43.4%) occurring in surgery using computer navigation assistance; 17 fractures were identified intraoperatively and 6 fractures were identified postoperatively either during immediate postoperative radiography or in subsequent follow up. These patients did not have history of further injury or trauma after surgery, thus they were assumed to be incurred during surgery and were included in this study. Of the 14 fractures with documented causes, 2 occurred during chamfer cut, 4 after box cut of the femoral implant, 2 during the trial of the femoral component, 5 during impaction of the final implant, and 1 during insertion of the polyethylene liner (Fig. 1).

**Table 1:** Summary of patients with intraoperative femoral condyle fracture

Patient	Sex	Age at operation (years)	Side	BMI (kg/m^2^)	Documented risk factor for Osteoporosis	ASA	Surgeon level	Model	Femoral Size	Navigation	Site of fracture	Displacement	Time discovered	Step of which fracture occured	Management	LOS (days)	Latest FU from primary OT (years)	KSS (knee) at latest FU	KSS (function) at latest FU
A	F	56	Left	32.1	–	2	Specialist	Legacy	E	No	Medial condyle	Nil	Postoperative	Unknown	Conservative	6	5	92	90
B	F	67	Right	26.4	–	2	Specialist	PFC	3	No	Medial condyle	Nil	Postoperative	Unknown	Conservative	8	4.75	94	90
C	F	81	Right	21.9	Age > 70	2	Specialist	Triathlon	1	No	Medial condyle	Nil	Postoperative	Unknown	Conservative	7	3.8	72	70
D	F	57	Right	28.6	–	2	Specialist	Legacy	C	No	Medial condyle	Yes	Postoperative 2 months	Unknown	Revision TKA	4	2.3	77	80
E	F	75	Left	24.7	Age > 70	2	Trainee	Triathlon	3	No	Medial condyle	Nil	Intraoperative	During PE insertion	Screw fixation	6	3.5	89	80
F	F	68	Right	29.7	CRF	3	Specialist	Legacy	E	No	Lateral condyle	Nil	Intraoperative	During trial of femoral component	Screw fixation	8	3.75	99	85
G	M	50	Left	30.1	–	2	Specialist	Legacy	F	No	Medial condyle	Nil	Intraoperative	Unknown	Screw fixation	6	3.75	100	90
H	M	65	Left	28.5	–	3	Specialist	Legacy	D	No	Medial condyle	Nil	Intraoperative	During impaction of implant	Screw fixation	11	3	98	85
I	F	74	Right	26.1	Age > 70	2	Specialist	Legacy	C	Yes, iAssist	Medial condyle	Nil	Intraoperative	Unknown	Screw fixation	6	3	99	75
J	F	64	Left	33.3	–	2	Specialist	Triathlon	4	No	Lateral condyle	Nil	Intraoperative	During impaction of implant	Conservative	5	2.75	90	75
K	F	77	Right	31.3	Age > 70	2	Specialist	Legacy	D	Yes, iAssist	Medial condyle	Nil	Postoperative	Unknown	Conservative	11	1.5	96	70
L	M	85	Left	20.5	Age > 70, History of fracture left hip	3	Trainee	Triathlon	3	Yes, ASM	Medial condyle	Nil	Intraoperative	During impaction of implant	Conservative	13	0.3	75	70
M	F	71	Right	24.3	Age > 70	2	Trainee	Triathlon	2	Yes, ASM	Medial condyle	Nil	Intraoperative	After box cut of femoral component	Screw fixation	9	5.9	91	85
N	F	66	Right	22.9	–	2	Specialist	Legacy	D	No	Lateral condyle	Nil	Intraoperative	After box cut of femoral component	Screw fixation	7	5.6	93	85
O	F	82	Right	22.5	Age > 70	2	Specialist	Triathlon	1	Yes, ASM	Lateral condyle	Nil	Intraoperative	After box cut of femoral component	Screw fixation	10	3	94	65
P	F	56	Right (Bilateral OT)	34.0	CRF	2	Specialist	Triathlon	2	Yes, ASM	Medial condyle	Nil	Intraoperative	After box cut of femoral component	Screw fixation	22	4.3	71	95
Q	F	72	Left	23.4	Age > 70	3	Trainee	Legacy	D	No	Medial condyle	Nil	Intraoperative	During trial of femoral component	Screw fixation	6	2.7	89	60
R	F	72	Left (Bilateral OT)	30.7	Age > 70, Breast cancer	2	Specialist	Triathlon	3	Yes, ASM	Lateral condyle	Nil	Intraoperative	Unknown	Screw fixation	10	0.3	78	80
S	M	58	Left (Bilateral OT)	27.5	–	2	Specialist	Triathlon	5	Yes, ASM	Medial condyle	Nil	Intraoperative	During impaction of implant	Screw fixation	5	2	85	90
T	F	81	Left	23.8	Age > 70, History of osteoporotic spinal collapse	2	Specialist	Attune	5	No	Lateral condyle	Nil	Intraoperative	During impaction of implant	Screw fixation	7	2	97	80
U	F	74	Both (Bilateral OT)	23.2	Age > 70	2	Specialist	Triathlon	2 (Both sides)	Yes, ASM	Medial condyle	Nil	Intraoperative	During chamfer cut (both sides)	Screw fixation	8	0.7	92	80
V	F	73	Right	26.4	Age > 70	2	Trainee	Evolution	2	No	Medial condyle	Nil	Postoperative 1 week	Unknown	Conservative	4	0.9	94	85

*F* female, *M* male, *LOS* postoperative length of stay, *OT* operation, *BMI* body mass index, *ASA* American Society of Anaesthesiologists Physical Status Classification, *iAssist* iASSIST Knee System, Zimmer, *ASM* eNdtrac ASM Knee Navigation System, Stryker, *DM* diabetes mellitus, *CRF* chronic renal failure with estimated glomerular filtration rate (eGFR) < 30 mL/min/1.73m^2^, *Triathlon* Triathlon® Knee System, Stryker, *Legacy* NexGen® Legacy® Posterior Stabilized (LPS) Flex Fixed Bearing Knee, Zimmer, *PFC* P.F.C.® Total Knee System, DePuy Synthes, *Attune* ATTUNE® Knee System, DePuy Synthes, *Evolution* EVOLUTION™ Medial-Pivot Knee System, MicroPort, *TKA* total knee arthroplasty, *FU* follow up, *KSS (knee)* Knee Society Knee score, *KSS (function)* Knee Society Function score

Patients’ demographics and implant/navigation systems were analysed using the χ^2^ test, Fisher’s exact test or Student’s *t* test (Table 2). There was a trend of a higher fracture rate in the women (*p* = 0.098) and in Stryker ASM navigation (*p* = 0.109). There was a significant increase in fractures among patients who underwent bilateral TKA in the same session (*p* = 0.031) and more specifically, bilateral surgery under ASM navigation (*p* = 0.014).

**Table 2:** Results

	Fracture (*n* = 23)	No fracture (*n* = 2659)	*p* Value (χ^2^/t test/Fisher’s exact)
Age (SD)	69.5 (9.4)	69.2 (7.7)	0.871
BMI (SD)	26.7 (3.9)	27.7 (4.3)	0.268
Gender (F%)	82.6%	68.0%	0.098
Side (L%)	47.8%	49.0%	0.910
Bilateral	5 (21.7%)	236 (8.9%)	0.031
Navigation			
ASM	8 (34.8%)	579 (21.8%)	0.109
Bilateral ASM	5 (21.7%)	208 (7.8%)	0.014
i-Assist	2 (8.7%)	244 (9.2%)	0.937
Bilateral i-Assist	0 (0.0%)	10 (0.4%)	0.917
Implants			
Triathlon	11 (47.8%)	992 (37.3%)	0.299
Legacy	9 (39.1%)	927 (34.8%)	0.669
PFC	1 (4.3%)	219 (8.2%)	0.499
Attune	1 (4.3%)	158 (5.9%)	0.747
Evolution	1 (4.3%)	74 (2.7%)	0.650

*SD* standard deviation, *F* female, *L* left, *iAssist* iASSIST Knee System, Zimmer, *ASM* eNdtrac ASM Knee Navigation System, Stryker, *DM* diabetes mellitus, *CRF* chronic renal failure, *Triathlon* Triathlon® Knee System, Stryker, *Legacy* NexGen® Legacy® Posterior Stabilized (LPS) Flex Fixed Bearing Knee, Zimmer, *PFC* P.F.C.® Total Knee System, DePuy Synthes, *Attune* ATTUNE® Knee System, DePuy Synthes, *Evolution* EVOLUTION™ Medial-Pivot Knee System, MicroPort, *TKA* total knee arthroplasty, *LOS* postoperative length of stay, *FU* follow up, *KSS (knee)* Knee Society Knee score, *KSS (function)* Knee Society Function score

**Fig. 2:**
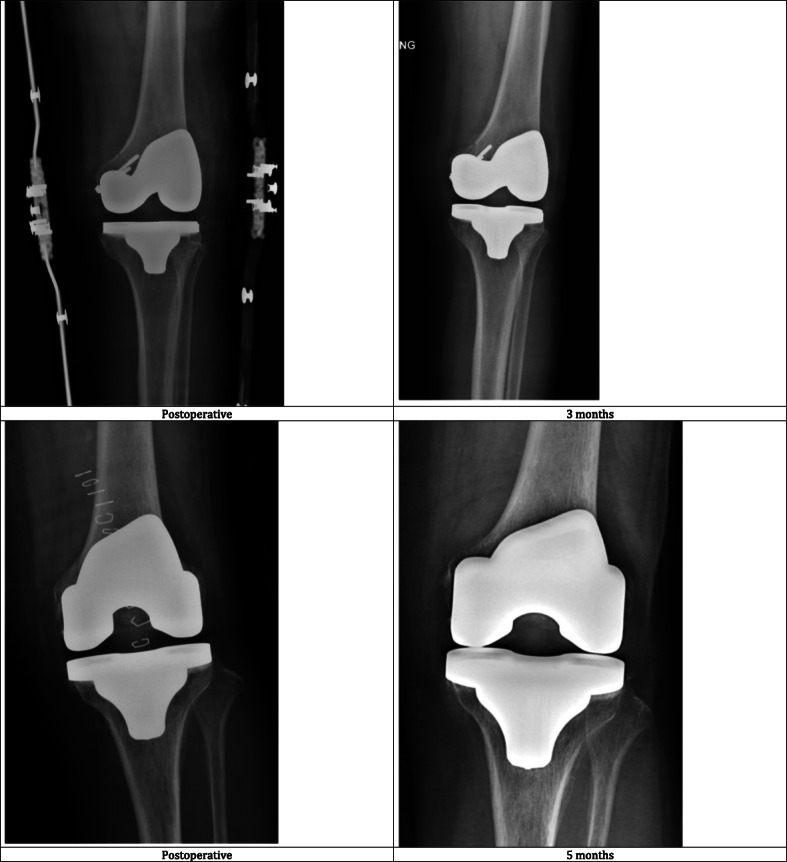
Postoperative radiographs of two patients who suffered from intraoperative femoral fracture

There were 7 patients managed conservatively with partial weight-bearing for 1–2 months, and 15 patients underwent immediate fixation with cannulated screws. Fracture status was assessed both clinically and radiologically during serial follow up. All patients achieved bone union (Fig. 2) and a good functional outcome. The mean Knee Society Knee score was 89.4 (range 71–100) and the function score was 80.2 (range 60–95) at a mean of 3.0 years post-operation.

One patient (Patient D; Table 1) required revision arthroplasty at 2 months after the index operation. Her initial TKA was performed uneventfully. However, during routine follow up she was noted to have slow rehabilitation progress and persistent knee pain. Varus deformity was evident on clinical examination. Radiography confirmed a displaced medial condyle fracture with femoral component loosening. The patient underwent revision of the femoral component. On subsequent follow up, there was clinical and radiological confirmation of fracture healing, and the patient was able to walk unaided with minimal pain.

## Discussion

To our knowledge, this is the first reported case-control study of intraoperative femoral condyle fractures during TKA performed in an Asian population. Clinical data have been scarce in describing the incidence and causes of such fractures. Alden et al. [1] reported an incidence of 0.39% for all TKA-related intraoperative fractures, with 73% of fractures occurring in the femur. Pun et al. [2] reported 5 femoral (0.37%) and 12 tibial (0.89%) fractures among his sample of 1345 TKAs. Pinaroli et al. [3] reported a 2.2% incidence of intraoperative fractures among 1795 TKAs, with only 25% of fractures occurring in the femur.

In our review, we report a 1.04% incidence of TKA intraoperative fractures in 2682 consecutive primary TKAs performed in a high-volume centre, with fractures occurring mostly at femoral condyle (82.1%). The overall incidence is compatible with other studies, while the cause of the higher occurrence of femoral condyle fractures may be multifactorial, as presented in Table 3.

**Table 3:** Postulated causes of intraoperative femoral condylar fractures in TKA

Patient factors	Implant/instrument factors	Technical factors
Osteoporosis Female gender Small size	Large/wide box cut in posterior stabilized TKA Pin-track positioning Computer navigation-assisted TKA (pin track)	Inadequate/excessive/eccentric box cut Eccentric/angular trial insertion or removal Excessive hammering force

*TKA* total knee arthroplasty

Patient factors play a part in these fractures. Osteoporosis is a known risk factor for periprosthetic femoral fracture following TKA [7]. Contrary to conventional belief, osteoporosis is now considered as common in Asian as in Caucasian populations [8, 9]. Low body weight, loss of weight, physical inactivity, use of corticosteroids or anticonvulsants, primary hyperparathyroidism, diabetes mellitus type 1, anorexia nervosa, gastrectomy, pernicious anaemia, and older age (> 70–80 years) are known to be predictors of high risk of osteoporosis [6]. Indeed, many of our TKA candidates who suffered from end-stage knee osteoarthritis were older and commonly had comorbidities. Of our 23 patients with femoral condyle fracture, 63.6% had at least one strong risk factor for osteoporosis. Nevertheless, due to the small number of fractures relative to the large total cohort, it was not possible to statistically delineate the relationship of these fractures with comorbidities and osteoporotic risk factors. Bone densitometry would be an objective indicator; however, it was not routinely performed in patients receiving TKA.

Alden et al [1] postulated female gender may be an important factor in intraoperative fractures. Postmenopausal women have a higher prevalence of osteoporosis and greater incidence of fracture than men of similar age [10], particularly in the femur. A recent morphological study in an Asian population has also confirmed that Asian women have a lower femoral width-height ratio than Asian men [11]. That is, for the same femoral anteroposterior dimension, women have a narrower femur compared to men. In the case of a posterior-stabilized implant, women would be left with narrower condyles after box cut compared to men of the same femoral size, predisposing to condylar fractures. Women have also been shown to have a smaller average femoral dimension than men [9]. In our study, there was a trend of a higher percentage of female patients having femoral condyle fracture (82.6 vs 68%, *p* = 0.098).

**Fig. 3:**
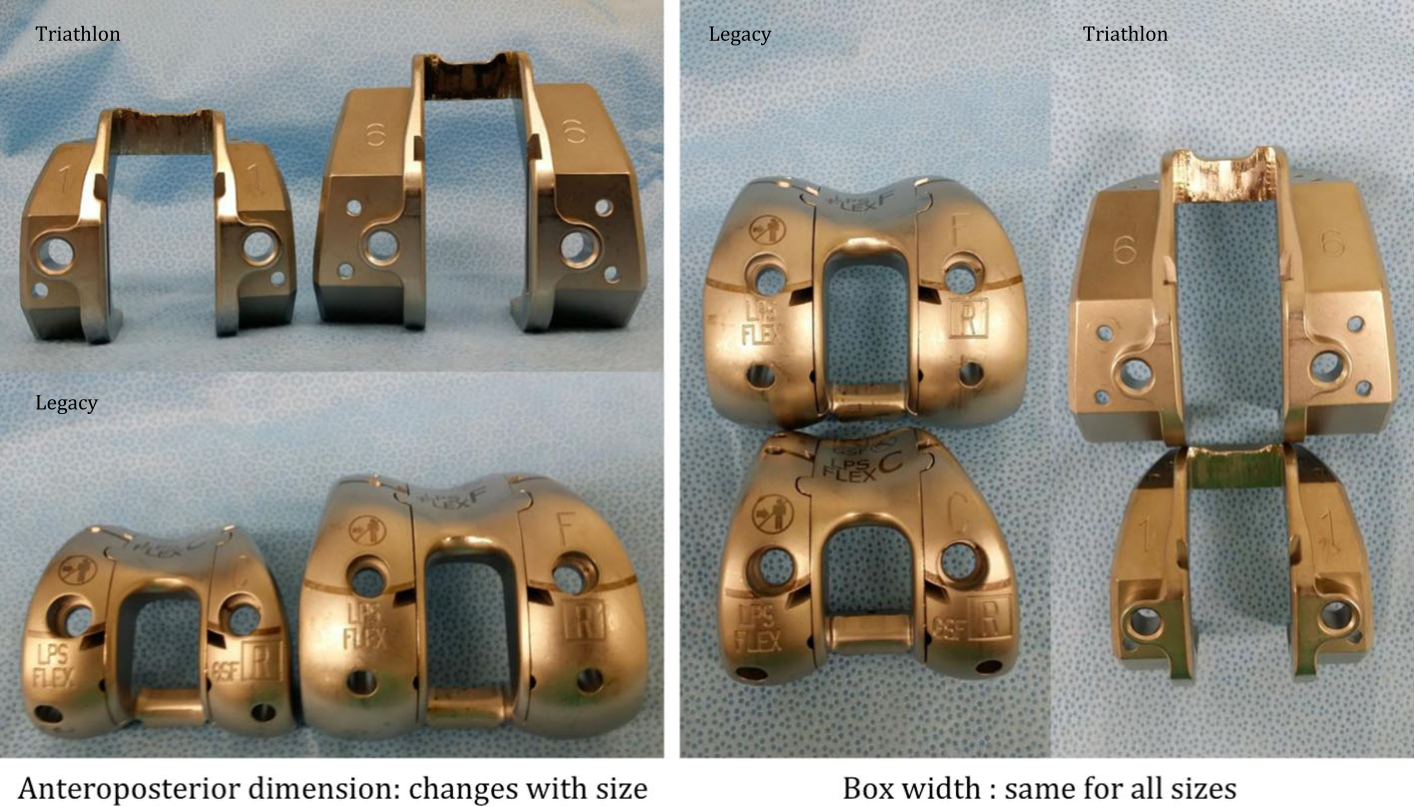
Box cut design of Triathlon® (Stryker) and NexGen® Legacy® (Zimmer) femoral components

Implant design may also have an important role. In posterior-stabilized implants, the intercondylar box cut poses significant technical challenges. Alden et al [1] reported an increased risk of femur fracture with posterior-stabilized implants compared to cruciate retaining implants (relative risk 4.74). Delasotta et al. [12] demonstrated that the width and depth of the box cut remained the same with different sizes of Triathlon® (Stryker) implants. It was consistent with our experience of both the Triathlon® (Stryker) and NexGen® Legacy® (Zimmer) implants (Fig. 3). In patients requiring a smaller sized femoral implant, the box cut was proportionally larger and more bone was resected in proportion to the total femoral volume, weakening the strength of both condyles. Indeed, amongst the four condylar fractures due to box cut that we identified in our study (Patients M, N, O, P; Table 1), smaller sized femoral implants were used coincidentally. The proportionately excessive box cut posed a genuine risk in these patients. Because of the smaller body build in Asian people, one would expect the chance of using smaller femoral sizes to be higher. This might explain the much higher percentage of femoral fracture in the present study than in older reports [1–3] (82.1% vs 25–47%).

**Fig. 4:**
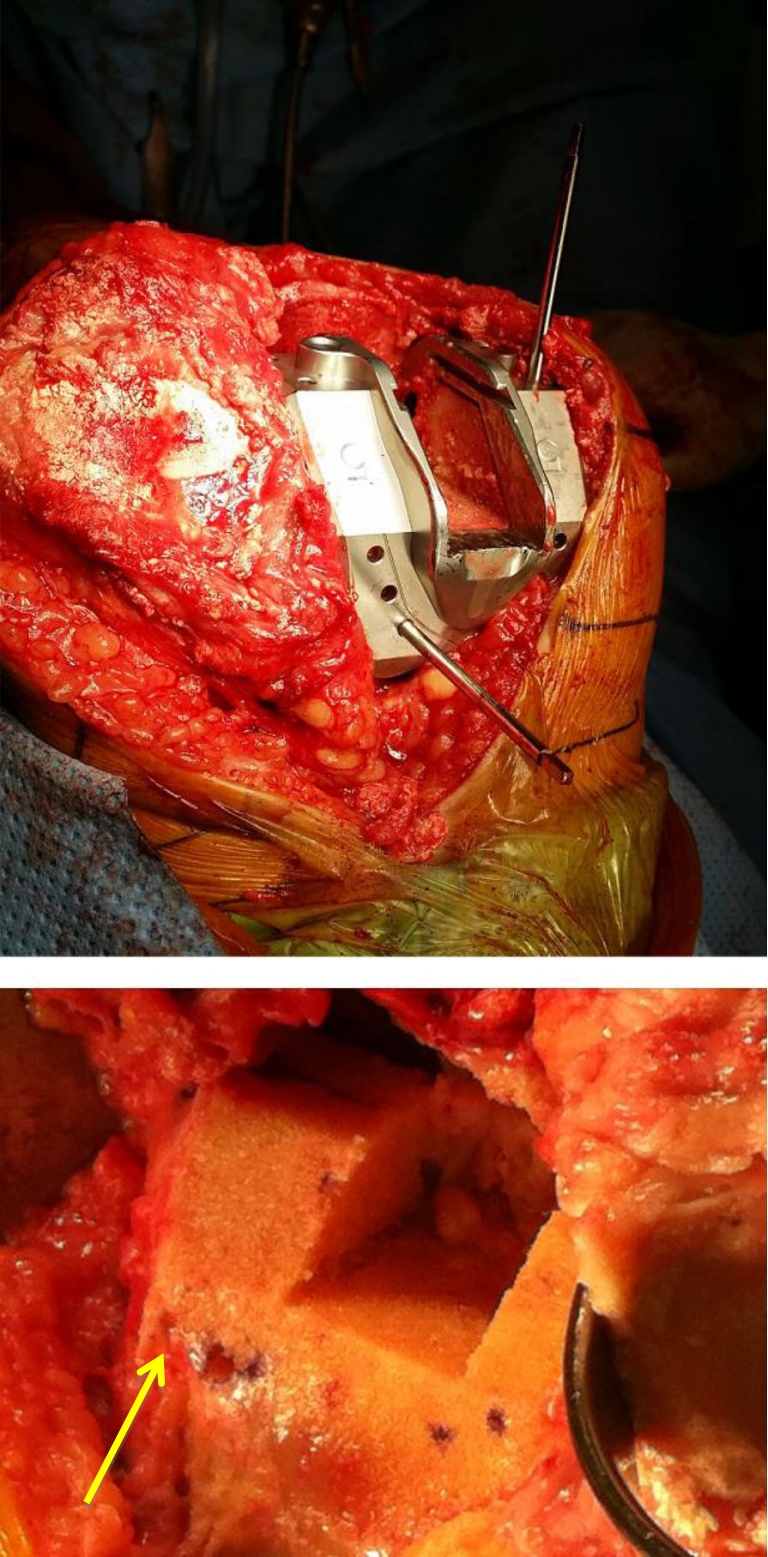
Pin tracks involved during (up) and after (down) femoral preparation for Triathlon® (Stryker) femoral components

**Fig. 5:**
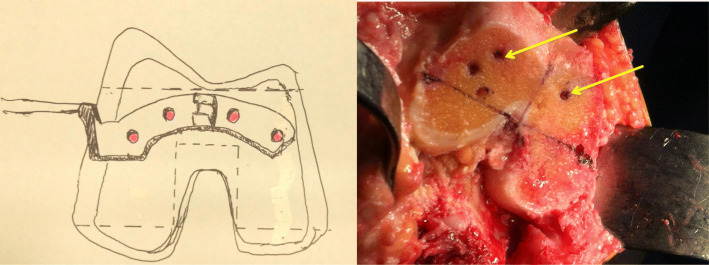
Computer navigation pin tracks on femoral condyle using the eNdtrac ASM Knee Navigation System (Stryker)

Pin tracks involved in temporary fixation of cutting jigs and sizing guides may further weaken the distal femur bone cut (Fig. 4). In our series, one patient suffered from bilateral medial condyle fracture due to the same mechanism - both fractures along the pin track of the cutting jig during chamfer cut. Pin-track-induced fractures were also reported in computer navigation-assisted TKAs. In our series, 10 fractures occurred with computer navigation. Weakening of the femoral condyle with the positioning of navigation-related pin tracks was observed (Fig. 5). Beldame et al. [13] suggested that suboptimal placement of pins in the diaphyseal femur, or transcortical fixation of pins were the likely causes of weakening and subsequent fracture. There was a mean delay between arthroplasty and fracture of 12.6 weeks, preceded by several days of thigh pain or occurring after a minor injury. The authors suggested bi-cortical metaphyseal fixation of tracker pins for computer-assisted TKA.

Several studies on the technical aspect of TKA in intraoperative femoral fractures have been published. A classic study by Lombardi et al. [14] demonstrated 40 intraoperative distal femoral intercondylar fractures in 898 primary posterior-stabilized TKAs (4.4%). The high incidence rate was attributed to inappropriate intercondylar box cuts, which were either not wide enough or were placed eccentrically, weakening the respective condyle. When they changed to a different implant with particular attention to the box cut with intercondylar sizing-guide verification, the incidence of intercondylar fracture dropped to 0.2%. Furthermore, Lombardi et al. [14] suggested angular or eccentric trial insertion or removal could cause stress to either condyle, increasing the risk of fracture. Ancillary devices were often designed in modern TKA systems to aid with insertion or extraction of trial and final implants.

In our series, most fractures occurred during impaction of the final femoral implant (Fig. 1). We postulate a multifactorial explanation of this phenomenon, in which an inadequate box cut, previous weakening of bone with a sub-optimally placed pin track, eccentric direction of hammering, coupled with excessive hammering force could cause fractures at this stage. Ideally, we suggest a prudent technique in trial and final implant insertion, where implants should be inserted half way by hand to confirm correct orientation and adequate dimensions of bone cut (especially in the box cut) before lightly hammering down into the final position.

One interesting finding in the present study was a significant increase in fractures among patients who underwent bilateral TKA in the same session and, more specifically, in bilateral surgery under ASM navigation. In our knowledge, this is the first study suggesting such a correlation. We postulate that the reason for the higher fracture rate in bilateral surgery is multifactorial. Firstl patients with more severe osteoarthritis in both knees tend to undergo bilateral surgery in our centre for one-stage symptom relief. These often require more complicated surgery and would therefore increase the risk of fracture. Second, 88.1% of bilateral surgery in our centre was performed using Stryker ASM navigation system. The demonstrated tendency to fracture inevitably confounds the result. Technical factors due to speedy surgery or surgeon fatigue may also contribute to the incidence of fracture. Further study is required to investigate the causative factors in intraoperative fracture with bilateral TKA.

Management options for femoral condylar fractures include conservative management, internal fixation or revision arthroplasty with stems and augments. Studies showed favourable results and prognosis after these fractures [1, 3, 12]. In our centre, stable fractures were treated conservatively with partial weight-bearing or fixed intraoperatively with cannulated screws according to the surgeon’s discretion. Amongst our series of 2682 consecutive primary TKAs, only one patient required revision TKA due to intraoperative fracture. All patients achieved clinically and radiologically confirmed healing, and they all had an excellent outcome.

A limitation of this study was the lack of objective evaluation of osteoporosis in our patients, as bone densitometry was not routinely performed in our locality. Another limitation was its retrospective nature. However, the low incidence of intraoperative fractures in TKA deemed a prospective study implausible.

## Conclusions

In conclusion, intraoperative femoral fracture in modern TKA is not uncommon in Asian populations, particularly in navigated and bilateral PS TKA. Extra care may be required in small female patients with risk factors for osteoporosis. The authors would stress the importance of choosing a PS implant with an adaptive box size in patients with small, osteoporotic knees. Nevertheless, a good functional result can be expected after proper treatment of femoral condyle fractures.

## Data Availability

All details of data, tables and figures are available through the corresponding author. Detailed data on patients involved are not available to the public due to protection of patients’ privacy.
